# Bioprospecting of gut microflora for plastic biodegradation

**DOI:** 10.1080/21655979.2021.1902173

**Published:** 2021-03-26

**Authors:** Sini Francis CF, Sharrel Rebello, Embalil Mathachan Aneesh, Raveendran Sindhu, Parameswaran Binod, Suren Singh, Ashok Pandey

**Affiliations:** a St. Joseph’s College, Irinjalakuda, Kerala, India; b Microbial Processes and Technology Division, CSIR-National Institute for Interdisciplinary Science and Technology, Trivandrum India; c Centre for Innovation and Translational Research, CSIR – Indian Institute for Toxicology Research, Lucknow India; d Faculty of Applied Sciences, Durban University of Technology, Durban, South Africa; e Centre for Energy and Environmental Sustainability, Lucknow India.

**Keywords:** Plastic bioremediation, microbes, petase, hydrolase, biodegradation, gut microbe

## Abstract

The problem of plastic prevalence and associated pollution has grasped the entire planet drastically, putting all fields of science on the stake seeking remedies to this global havoc. To address this crisis, with a single remediation strategy is often found to be baseless, thereby much interest has been evoked in the development of multidisciplinary approaches – involving physico-chemical and biological strategies to nullify the aftermath of plastic pollution in all possible means. Even amidst, the availability of different approaches, the use of biological methods to combat plastic degradation has gained momentum. The most frequently used plastics appear in wide forms such as polyethylene plastic bags, polypropylene-based bottles, polyvinyl chloride pipes and polystyrene styrene cups. Plastic nicknamed as one of the toughest polymers viz. polycarbonate, acrylonitrile butadiene styrene (ABS) and Polydicyclopentadiene; quite often are called so as they resist degradation in normal environmental strategies. They are often degraded in non-hostile and harsh environments of pH, temperature, radiation etc. However, not always it is possible to create such harsh environments for plastic degradation. In such a scenario, the use of gut microbes that can withstand the harsh atmosphere of gut environment could serve as promising candidates for plastic biodegradation. The current article envisages the various gut microbes of various biological agents and their role in plastic remediation. The current review compiles the techniques available for plastic remediation, the microbial prospects of plastic remediation, its challenges, and possible breakthroughs to effective plastic remediation.

## Graphical Abstract

**Figure:**
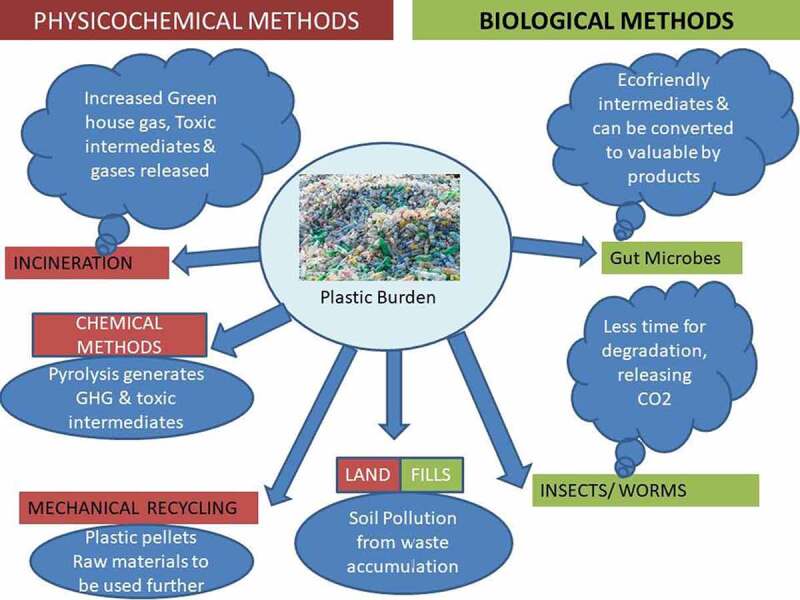

## Article Highlights

- An overview of bad effects of plastic pollution.
- Strategies to tackle plastic pollution.
- Regulations to combat plastic pollution.
- Role of gut microbes in plastic remediation.
- Gut microflora promising candidate for plastic degradation.

## 1. Introduction

Plastic is a versatile polymer, popular for its wide utility, flexibility in synthesis, and consumer-satisfying properties finding applications in almost every industry. The properties of low electrical conductivity, low density, and transparency make it perfect for various applications [1]. The use as packaging material, storage containers for various commodities, plastics are available in different types, composition, durability and recyclability [2]. The use of plastics in medical devices, drug delivery devices and packaged drugs are also quite prevalent [3]. Even the occurrence of degradation-resistant plastics such as polycarbonates and ABS do find their way into industrial and household applications. Apart from this an era of conductive plastics also has emerged with applications in electronic industry [4,5].

Regardless of their versatile use, the environmental problems and associated health issues induced by plastics in human beings are growing day by day. The increased incidents of birth defects, cancers, poor immunity, defective reproductive health among human community is highly correlated with our frequent exposure to plastic and plastic products [6]. (Table 1) depicts the toxicity of plastic polymers to human health. A comparative analysis on the effect of various plastic extracts particularly of polyvinyl chloride (PVC) and polyurethane (PUR) exhibited maximum baseline toxicity, oxidative stress and estrogenicity [7]. The production of plastics such as PVC are noted for their carcinogenic potential, exposure of toxic chemicals like dioxins to workers and people living in contaminated localities (Vinyl Chloride (saferchemicals.org).

**Table 1.** Harmful effects of different plastics against human and environment

Type of plastic	Monomers used in polymerisaton	Use of specific plastic	Health Issue
Polyethylene	H_2_C = CH_2_	Cosmetics, adhesive, emulsion stabilizer, film former, an oral care agent	Endocrine disruptors [11], reproduction [12], Chromosome instabilities in human lymphocytes [13]
Polypropylene	(C_3_H_6_)n	Consumer products, automobiles, packaging etc	Respiratory disorders [14], large sized particles affected immune system and induced hypersensitivity [15]
Polyvinylchloride	(C_2_H_3_Cl	Pipes, children toys, chewy teethers, luggage, backpacks with shiny plastic designs, automobile parts etc	human carcinogen, toxicity to every major organ system,
Polystyrene		Protective packaging, containers, lids, bottles, trays, tumblers etc	Harmful to Central nervous system
Polytetrafluoroethylene (Teflon)	CF_2_ = CF_2_	Nonstick surfaces, plumbing tape, chemical-resistant containers and films	Causes tumors and neonatal death and may have toxic effects on the immune, liver, and endocrine systems [16].

Apart from the plastic polymers, various chemicals associated with plastic also induce a high risk to health of consumers as well as workers involved in plastic production. The health hazards induced by plastic toxicity are mainly caused by migration of plastic associated phthalates and other resident chemicals to food as well as water of human diet [8,9]. Phthalates often used as plasticizers are potent endocrine disruptors critically affecting the male fertility and child health [10]. Though PET-based water bottles are safe the usage of PET bottles for storing or serving low acid drinks would release phthalates and heavy metals antimony from the bottle [6].

More than 70% of polymers produced around the world in each year are released directly into the environment which get deposited in the soil as landfills or enter into the marine habitat due to the lack of proper disposal methods. Statistical analysis of plastic disposal indicates that in 2020 almost 8 to 14 million metric tons (Mt) of plastic waste found its entry into oceans without any degradation methods adopted (100+ Plastic in the Ocean Statistics & Facts (2020) (condorferries.co.uk). Most of the industrial waste released from industries reach the rivers and drainage system and finally in seas and oceans [17].

Macroplastics are reaching the marine habitat which is released as a part of fishing, shipping, aquaculture, and the tourist industry. It may be converted into tiny secondary microplastics by the activity of abiotic factors like UV radiation, temperature, oxygen, and other physical means [18] by which it will be able to reach other geographical areas including marine ecosystem. Chemicals such as Bisphenol A, Phtalate, Polychlorinated biphenyls, polycyclic aromatic hydrocarbons (PAHs), organochlorine pesticides (OCPs), polybrominated diphenyl ethers (PBDEs), alkylphenols etc components in plastic do reach the water bodies [19]. Such changes in marine water due to human activities has created a new plastisphere niche occupied by various organisms on the surface of plastics [20], simultaneously causing detrimental effects on formerly resident fauna and flora [21]. Plastics can be a housing for various creatures, both on the surface and deeper conditions of the sea as it sinks down [22].

Plastics pose both, direct and indirect hazards to the environment (aquatic and terrestrial) and inhabitants due to their consumption by the organisms and their entanglement in those substances, especially juveniles [23]. The main reason for the accumulation of plastics in their gut is their inability to regurgitate the ingested particles, chiefly in Procellariiformes and they are more susceptible in these conditions [24]. Delayed ovulation, decreased secretion of gastric enzymes and steroid hormones are the other impacts [25]. Some of the species like turtles feed on plastics due to their resemblance to their prey. The gut contents of 60% of sea turtle (*Chelonia mydas*), an endangered species contained the presence of plastics [26]. Plastic debris accumulated in the benthic zone of ocean, may interfere with the exchange of gases, leading to anoxic or lower oxygen conditions. Its negative impacts are on the marine organisms of these regions and thereby altering the species composition [27]. The organisms may derive toxic chemicals from the plastic debris which have detrimental effects on different species including man on entry into the food chain [28]. Thus, the remediation of plastic is the need of the hour.

The current review addresses the various possible methods to tackle the plastic pollution. A brief note on the currently used techniques and the prospects of biological methods in plastic remediation has been presented. The paper tries to present the regulations worldwide in this issue and the necessity to increase the research in the direction of biological strategies to tackle plastic pollution

## 2. Tackling plastic pollution

The problem of plastic pollution is addressed worldwide by adopting strict regulations to discourage the use of plastics and many nations have banned the use of single-use plastics [29]. Treaties such as MARPOL of 1970 (prevent ships dumping plastic waste in oceans), United Nations Convention on the Law of the Sea (UNCLOS) of 1982 (prevent dumping waste at sea), the Stockholm Convention on Persistent Organic Pollutants of 2001 (discourages use of harmful chemicals in plastic), the Basel Convention on the Control of Transboundary Movements of Hazardous Wastes and their Disposal of 2019 depict the continuous effort to combat the menace caused by plastics effectively and wisely [30]. Attempts to consider the issue of plastic pollution in the Paris Convention has achieved much attention in many participating countries. The Chinese ban on imported plastic products has greatly influenced to reduce the Plastic involved International Trade and subsequent plastic pollution [31].

The ban of single use plastics in India is still at stake amidst this global pandemic, in spite of India being the fourth largest producer of plastic waste among the whole world [32,33]. The plastic ban in several States across India have resulted in the development of various bioplastic alternatives [34]. Different strategies such as high payment for plastic bags, replacement of plastic bags by cloth bags or paper bags have been followed with the intention of reducing plastic waste across India. The Plastic Policy implemented in India lays out many regulations prohibiting use of recyclable plastic carry bags for Ready to eat drinks or food stuff, prevention of carry bags less than 50 microns etc https://www.enhesa.com/resources/article/new-plastic-waste-rules-in-india/). The efforts under the Swach Bharat Mission have taken measures to recycle plastic by mechanical means, feedstock cycling along with attempts to convert plastic waste to roads and sometimes toilets [35]. Regardless of the these multifaceted efforts to reduce plastic pollution, a huge heap of plastic waste is generated daily and the process of plastic disposal can be done mainly by three methods, recycling, incineration and dumping in landfills [36].

### 2.1. Recycling

The three principles of reducing, reuse and recycle though practiced do not provide true solutions to the burden of plastic waste remediation [37]. As per an evaluation discussed in Chemical Engineering News the ratio of different types of plastics recycled in America against their rate of production was found to be varying with 19.5% of 4.5 billion kg of Polyethylene thalate (PET), 10.3% of 5.5 billion kg of High density Polythylene (HDPE), 0% of 0.9 billion kg polyvinyl chloride (PVC), 5.3% of 7.4 billion kg low density polyethylene (LDPE), 0.6% of 7.2 billion kg polypropylene (PP), 0.9% of 2.2 billion kg polystyrene [38].This clearly shows the fact that majority of the plastic produced is left behind untreated or recycled. Plastics also differ in their recyclability as HDPE is harder than LDPE and thus can be easily passed through recycling machine; whereas LDPE being softer get associated with recycling machinery [39].

Even developing countries like India, have taken initiative to recycle plastics as noted by an through a digital network Rethink+ by Dow Chemical International Pvt. Ltd., Mumbai in collaboration with Recykal (https://www.recyclingtoday.com/article/dow-india-launches-rethink-plus-recycle-plastic-scrap/). Yet other initiatives of converting g post-consumer recycled (PCR) plastics to polyethylene films (https://www.waste360.com/recycling/dow-and-lucro-launch-pcr-plastics-solution-india) are also noteworthy projects to combat plastic pollution. Researches in the direction of converting plastic waste by pyrolysis into fuels are also open doors to chemical conversion of plastics to diesel at the Indian Institute of Petroleum, Dehradun (https://www.iip.res.in/waste-plastics-conversion-process-technology/dr-ajay-kumar/). Recycling polystyrene plastics with orange peel extract to form textile fabrics with ability to quench oil spills has also gained much attention due to its efficiency in remediating two xenobiotics plastic as well as oil [40].

The recycling process also has its own limitations as common people are not aware of plastics that are thrown away even after a single use and stabilizers and other coloring agents used, make the procedure ineffectual. Moreover, quite often plastic bottles and utensils get soiled or dirtied by food remnants requiring additional treatment methods.

### 2.2. Landfills

Dumping in landfills is not a proper way to get rid of plastics as are spoiling the area which can be otherwise used for any other purposes such as the cultivation of crops and the anoxic conditions in landfills also resist the natural process of degradation.

### 2.3. Incineration

While considering incineration, the end products released at last causes environmental pollution [41]. Plastic waste on incineration release large amount of toxic xenobiotics such as Dioxins, Furans, Mercury and Polychlorinated Biphenyls into the environment [42]. These toxic compounds impart negative effects to animal, plant and human health. In such a scenario, use of microbes for plastic degradation will prove a good strategy.

## 3. Microbes in plastic degradation

Microorganisms are ideal candidates for decontamination purposes as they have the capacity to synthesize enzymes and due to their small size, they get access to contact with the complete surface area. They use plastic and other environmentally harmful chemicals as a source of nutrients (carbon) and energy (electrons) [43]. The end products of degradation will be water and carbon dioxide along with the multiplication of microbial population [44].

Polyethylene being the mostly used plastic; reduction in PE (polyethylene phthalate) would bring a great impact on remediation of plastic waste. Results indicate the potential of biodegradation of plastic waste with selected microbial strains became a viable solution [45]. Various microbes such as *Brevibacillus borstelensis, Rhodococcus ruber, Ideonella sakaiensis, Serratia* sp. etc are found to degrade polythene-based plastics [46]. Polyethylene degradation by microbes alone has been done worldwide, to achieve approximately 20% degradation in 30 days period. Successful attempts in the Wax moth (*Galleria mellonella*) aided fast bio-degradation of PE was reported to generate ethylene glycol [47]. This also signifies the use of plastic-eating worms in remediation of plastic [48]. (Table 2) enlists various microbes used in the degradation of plastics.

**Table 2.** Plastic degrading microbes

Type of polymer	Type of Microbe	Genus	Remarks	Reference
Polyurethane	Fungi	*Aspergillus, Paecelomyces, Penicillium, Alternaria*, and *fusariumtrichoderma DIA-T* sp.	Most of the strains showed more urease and protease activity	[49]
Polyethylene	Bacteria	*Brevibacillus, Pseudomonas*, and *Rhodococcus*	*Pseudomonas* showed biodegradability of 40.5%	[50]
Polythene and plastic	Bacteria	*S. aureus, Micrococcus, S. pyogenes, P. aeruginosa* and *B. subtilis*	Experiment was done in fadama soil (fs) amended with poultry droppings, cow dung and inorganic fertilizer (npk)	[51]
”	Fungus	*Aspergillus niger, A. flavus, A. fumigates, Mucor, Penicillium* and *Fusarium*	”	[51]
Polyurethane	Fungus	*Pestalotiopsis*	Endophytes are isolated from wooden plants. enzyme belonging to serine hydrolase family is present	[52]
Low density polyethylene	Fungus	*Aspergillus* and *Fusarium* sp.	Depolymerization of polymers by the microbial enzymes.	[53]
Low density polyethylene (LDPE)	Fungus	*Aspergillus versicolor* and *Aspergillus* sp.	From marine water	[54]
LDPE	Bacteria	*Microbacterium* sp. *Pseudomonas putida* strain, *Pseudomonas aeruginosa, P. putida, P. aeruginosa*	Combination of potential bacterial strains accelerates degradation	[55]
Polyethylene	Bacteria	*Pseudomonas* sp.	*Pseudomonas* from sewage sludge dump showed high degradation potential (46.1%)	[56]
Disposable polyethylene	Bacteria Fungus	*Streptomyces* sp.,*Aspergillus flavus* and *Mucor rouxii* 1835	Fungus showed more degradation ability	[57]
Low density polyethylene	Fungus Bacteria	*Aspergillus niger* and *A. flavus* *Pseudomonas* sp,*Streptomyces* sp	*Streptomyces* sp showed high degradation capacity (46.7%)	[89]
Polythene	Bacteria Fungus	*Bacillus subtilis,, Staphylococcus aureus.,Streptococcus lactis, Proteus vulgaris, Micrococcus luteus* *Aspergillus niger, Aspergillus nidulance, Aspergillus flavus, Aspergillus glaucus, Penicillum species, Pseudomonas* sp.		[58]
Polyethylene	Bacteria	*Rhodococcus ruber*	Rhodococcus ruber a biofilm forming bacteria as it is highly hydrophobic(0.86%/week)	[59]
Thermo-oxidized (80°C, 15 days) low‐density polyethylene (TO‐LDPE)	Fungus	*Aspergillus niger* and *Penicillium pinophilum*	Thermo‐oxidized) low‐density polyethylene (TO‐LDPE) showed high degradation by the fungus when treated with ethanol as co substrate	[60]
High density polyethylene	Fungus	*Aspergillus niger, Aspergillus flavus, Aspergillus oryzae*	Exposure to uv radiation before inoculation of fungi increased the rate of degradation	[61]
Polyethylene	Bacteria Fungus	*Pseudomonas* sp.,*Bacillus* sp.,*Staphylococcus* sp., and *Streptomyces* sp. *Aspergillus nidulans, Aspergillus flavus*		[62]
Low density polyethene and polypropylene	Bacteria	*Pseudomonas stutzeri*		[63]
High-density polyethylene (HDPE)	Fungus	*Arthrobacter* sp. and *Pseudomonas* sp.	Soil from Marine habitat	[64]
Branched low-density (0Æ92 g cm)^3^) polyethylene (LDPE)	Bacteria	*Brevibacillus borstelensis*	Carbon (mannitol free medium)deprivation enhances the degradation process	[79]
Polyethylene	Bacteria	*Bacillus cereus* strain ma-su	Pretreatment with UV and thermal oxidation enhances biodegradation	[65]
Low-density polyethylene (LDPE)	Bacteria	*Staphylococcus epidermis*		[66]
Polylactide	Fungus	*Amycolatopsis* sp.		[67]

### 3.1. Gut microbes in plastic degradation

**Figure 1.**
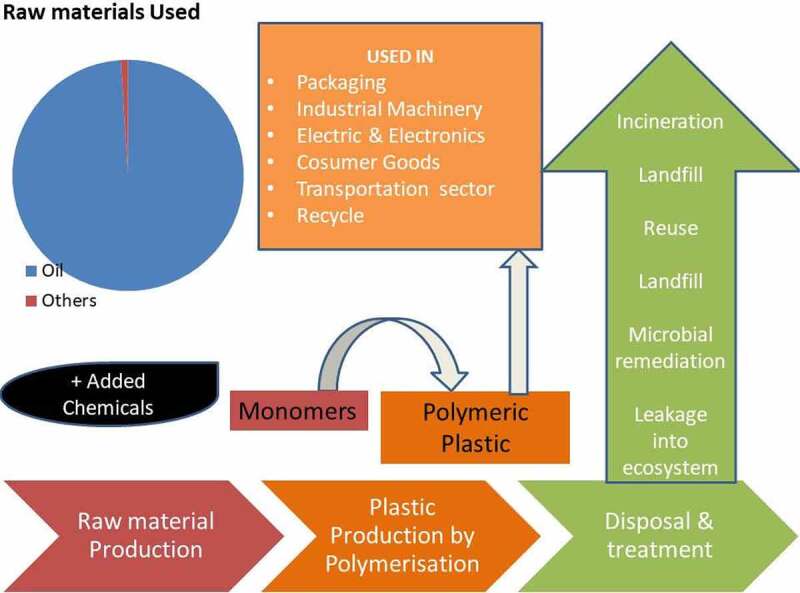
Schematic representation of the life cycle of plastic production to disposal

Microorganisms can thrive in extreme environmental conditions and bear many properties which will be useful for various macroscopic insects to withstand adversities created by various pollutants like plastics [68]. The gut environment of insects host a limited number of microbes compared to mammals, yet the ones prevalent are found to be promising to the host conferring in them properties to explore some rare nutritive factors, protection against pathogens as well as modes to elicit an immunological response [68]. The ability of greater wax moth *Galleria melonella* to naturally utilize beeswax as its food consisting of a mixture of alkanes, alkenes, fatty acids added an extra advantage to utilize PE which has similar CH_2_- CH_2_ – structures as bee wax [69]. The degradation of plastic by wax moth has been visualized in (Figure 1). Novel bacterial strains viz. *Myroides albus* sp [70]. and *Intestinirhabdus alba* [71] were isolated from the gut of *Zophobas atratus*a coleopteran larvae with plastic eating properties [72]. The discovery of yet more microbes in insect guts with plastic degradative ability is yet another proof the gut microflora has been evolving according to the prevalence of recalcitrant xenobiotics like plastics [73]. Furthermore, the ability of microbe *Pseudomonas aeruginosa* isolated from the gut of plastic eating super worms to digest a wide variety of plastics is yet another substantiating role of gut microbes in plastic degradation [74].

Larva of *Plodiainter punctella* (Indian meal moths or wax worms) was reported to capable of chewing and eating polyethylene. Yang and his co-workers, 2014 have isolated 2 bacterial strains from the gut which enable the digestion of polyethylene and thereby the wax worms could derive nutrients [75]. *Enterobacter asburiae* YT1 and *Bacillus* sp. YP1 were identified as the bacterial strains by culturing the dissected gut contents in a medium containing 1.0 g of the small PE pieces and 80 mL of LCFBM.

Mealworms (larva of *Tenebrio molitor* Linnaeus) also harbor polystyrene degrading microbes in their midgut. Polystyrene is treated as a non-biodegradable plastic [76]. The role of gut microbes in degradation can be explained and proven by analyzing the degradative ability after suppressing the gut microbes by using antibiotics. Studies also indicate that Styrofoam feeding of by edible insects such *Tenebrio molitor* and *Zophobas morio* not only increased their protein content but also exhibited little cytotoxic properties [77].

*Exiguobacterium* sp. strain YT2 was identified as the one able to cause changes in topography of surface, decrease the hydrophobicity and depolymerization of Styrofoam [78]. The bacteria was more efficient in depolymerization when it was inside the gut (47.7% inside the gut and 7.4% in 60 days outside medium) than in the medium, indicating that certain factors are favorable inside and only culturable bacteria was isolated and unculturable species remains unknown, that may be having a synergistic effect along with the other gut enzyme secreted by the larva itself [78].

*Brevibacillus borstelensis* is a thermophilic bacterium which could utilize polyethylene as a sole carbon source and reduced the molecular weight by 30% in the presence of mannitol [79]. Low density polyethylene (LDPE) is degraded by gut microbes of earthworm *Lumbricus terrestris* (Oligochaeta) and it is a powerful tool for soil restoration [80]. Biodegradable plastic degrading enzymes were characterized from two yeast strains collected from the larval mid gut of stag beetle *Aegus laevicollis* [81]. The commercial value of plastic degrading microbes greatly relies on their efficacy to degrade plastic in less time, further research and scale up studies to enhance their utility in plastic remediation further

### 3.2. Methods to study microbial plastic degradation

Various methods are used to analyze the extent to which a plastic polymer is degraded and how its quantification can be done both in laboratory conditions and in a natural environment. Morphological observation of plastics visually involving changes in the color, fragmentation, presence of fissures or holes are some manifestations of plastic remediation visualization as a preliminary step [82]. The process of visual evaluation often may not give quantitative results and thus it can only be used as a screening technique to identify plastic degrading microbes. Yet another strategy is by observing the formation of biofilms on plastics from the deposited site, either from an aquatic condition like inside the marine water or outside in the terrestrial conditions in soil or landfills [83]. The formations of biofilms are sometimes just an indication of microbial growth and not always, attachment of microbes to plastic will indicate a positive plastic degradation ability of microbes. The availability of different methods to assess plastic degradation is mainly to screen the microbial ability to degrade plastic. Additional pre-treatment of plastic samples are not needed to assess the rate of degradation.

Visual observations will not enable us to study a correct evaluation of how much the degradation has occurred. But it gives a primary hint that, the process is started. Atomic force microscopy (AFM) or scanning electron microscopy (SEM) were used in previous studies to get a clear picture by visual observations, differential scanning colorimetry (DSC), X-ray photoelectron spectroscopy (XPS), contact angle measurements and water uptake, Fourier Transform Infrared spectroscopy (FTIR), (NMR), X-ray diffraction (XRD), Nuclear Magnetic Resonance spectroscopy are some of the sophisticated techniques used for visual analysis and structural characterization [84–87]. FTIR Analysis or FTIR Spectroscopy can be used for observing chemical changes in the structure of various polymers as it will reveal the changes like formation and the disappearance of new chemical groups, formation of branches or debranching, addition of antioxidants, unsaturation etc in a study by [88] reported the loss of CHO stretching vibration and formation of a new peak at 939 cm^−1^ (O–H bend) in *Bacillus amyloliquefaciens* (bsm-1) and *Bacillus amyloliquefaciens* (bsm-2) treated low density polyethylene when compared with the control which is a clear indication of depolymerization. FTIR spectroscopic analysis is the best method in analyzing the microbial and enzyme degradation as it will provide a correct result about the formation and the disappearance of functional groups and chemical bonds. The use of techniques such as SEM and AFM enables a conclusive evidence for the plastic degradation ability as microscopic evidence is available; whereas techniques such as FTIR enable to obtain chemical evidence to microbial induced plastic degradation.

Weight loss measurements can be made to evaluate the percentage of degradation and it is widely used in numerous studies. Weight of the polymer sheet will be taken before and after inoculating with the microbe. The loss of weight can be calculated by the formula that, (initial weight – final weight) X 100/initial weight [89]. But various factors like adherence of cells and debris to the polymer due to improper washing may lead to a different result. This method is also known as liquid shaking culture test method [90]. pH changes can also be used as an indication of metabolic changes in microbes and enzymes [91]. The detection of microplastics in marine environments can also be visualized by staining with lipophilic dyes such as Nile Red [92].

When considering enzymatic and microbial degradation, significant changes may not occur in short periods, so that it will be useful for assessing physical degradation processes like bio-deterioration by UV light exposure and oxidation which causes considerable changes in its mechanical properties [93]. Under aerobic conditions, the degradation will result in an end product like carbon dioxide and oxygen will be consumed by the microbes. Different techniques have been developed to determine the concentration of CO_2_ starting from conventional methods like trapping of CO_2_ in Ba(OH)_2_ solution, followed by manual titration to infrared-gas analyzers. When the speed of reaction is very low, that is when the microbe is acting very slowly, the changes in concentration of these gases will also be very low.so that the determination of concentration should be done continuously with short intervals precisely in an accurate manner, which makes it a tedious task [94]. Stum test is also followed for the determination of evolved CO_2_ [88]. Use of radiolabelled CO_2_ gives a more precise result when used along with scintillation counter as it will not be interfered with biodegradable impurities and additives present in the polymer [95]. Controlled composting test is also utilizing the principle of release of CO_2_ during the process [96].

Agar-based visualization of degraded polymers in culture plates have also been practised. The formation of clear zones shows that they can not grow by utilizing the polymer as a nutritional and energy source [97]. It will be helpful in identifying the polymer degrading bacteria (which will grow in that medium) and can also be taken as a confirmation test even though the quantification of deterioration will not be possible.

## 4. Factors influencing microbial plastic degradation

Time is a major concern while considering the biodegradation of plastic as previous studies have reported that when examined the debris in marine water after a period of 3 weeks of incubation inside the water, biofilm was started to form, but there was no existence of plastic degrading bacteria, though its hydrophobicity was decreased [17]. Most of the degrading bacteria reach the surface and consume the polymer, only after a long time period. A previous study by Artham and his colleagues conducted in Bay of Bengal also showed that the productivity of surrounding marine ecosystem is also a determining factor of biofilm formation [98]. The environment and its diversity also influence plastic remediation as noted in a comparison of specific surface degradation rate of different kinds of plastics like HDPE and Polylacticacid (PLA) at marine environments and on land [99]. Results in the above study indicated that specific surface degradation rate of HDPE and PLA at marine environments were almost same of 11 μm year^–1^, whereas the degradation rate of PLA was 20 times higher than HDPE on land. Though the influence of microbes was not discussed in the above paper, the variability in the microbes of the soil environment and marine environment can be suggested to be a major contributory factor to such variation.

To generalize the factors affecting microbial degradation of plastics would be impractical as plastics are polymers with high level of variability with many additives which further adds more complexity to its structure. Chemical and physical properties of plastic is a major factor that will determine the way in which and rate at which, it will be degraded. Elasticity, hydrophobic nature, molecular weight, crystallinity, the type of functional group present in its structure, and colorants or additives added to the polymer, all play an important role in its breakdown [100].

Plastic particles must be assimilated into the microbial cell for the enzymes to get access. Low molecular weight is good for this assimilation step, as high molecular weight will decrease the solubility [101]. For example, polyethylene causes considerable damages to the environment as it has high molecular weight and hydrophobicity making the degradation process more difficult [102]. UV radiation is a worthy factor which will destroy the plastic very efficiently. As the exposure to UV radiation increases, the degradation rate also increases by the production of hydroperoxides by initiating the oxidative process [103]. Transferring of polyethylene into a medium containing *Fusarium* sp. Af4 after pretreating with UV and nitric acid have increased the rate of degradation while comparing with the control containing polyethylene without pre-treatment [104]. Increased temperature will also favor degradation as chemical reactions proceeds faster as temperature increases [105]. Pressure will be high at deeper parts of sea which will make the plastics, smaller fragments and fasten the deterioration even though the temperature and light penetration is low [106]. Process of weathering action of waves also have impacts on surface colonization [107].

## 5. Enzymes and challenges in microbial plastic degradation

A close evaluation on microbial degradation of plastics indicates that they do need longer incubation time to give considerable results. The main bottleneck to this problem is the meager quantity of degradatory enzymes such as depolymerases, hydrolases, and peroxidases produced by plastic bioremediating microbes [108]. Various enzymes of microbes such as laccases, esterases, lipases, cutinases, hydrolases etc contribute to plastic degradation [109]. For instance, the hydrolase and tannases of recently isolated PET degrading bacteria *Ideonella sakaiensis* that enable it to degrade PET by their hydrolytic action, disulfide bond removal ability etc [110].

The studies worldwide are still at a stage of identifying long list enzyme cocktails candidates which could prove promising in the direction of plastic degradation. The development of Polyethylene tetraphthalate (PET) degrading plastic degrading two-enzyme system from *Ideonella sakaiensis* to generate soluble mono(2-hydroxyethyl) terephthalate (MHET) eventually degraded to ethylene glycol and terephthalic acid is yet another progress in the biodegradation of plastic [111]. Modification of PET degrading polyesterase has been done to increase its action on PET in the above study, however, more advancements are yet needed in this direction. Thus, research should be further directed to increase the yield of such plastic degrading enzyme cocktails- following the principles of fermentation technology-based scale up using *I. sakaiensis* or even molecular tools that could induce the production of these degrading enzymes. The production of recombinant cutinases for butyrate production with improved yield is another example of such a case [112] and the extension of such principles for the purpose of plastic degradation would aid to find better solutions in near future. The chaperon induced expression of yet another thermostable pET hydrolyzing enzymes with a melting temperature as high as 101°C has been recently reported in *Bacillus subtilis* with an expression rate of 0.66 g/L [113]. Thus continuous research in the direction of plastic bioremediation could surely lead to its increased application in plastic remediation.

## 6. Conclusions and future prospects

The key factor to address the plastic menace is to reduce the use of plastic as much as possible and if used more preference to choose biodegradable plastic than recalcitrant counterparts. Plastic bioremediation using microbes is yet at a stage of infancy and needs to be developed to be commercialized and well-organized use of these microbes along with physical and chemical methods will be also helpful to get rid of plastic in the near future. The scope of microbial biodegradation of plastics would be more effective if it is replaced by the concept of microbial bioconversion of plastics to valuable products as the degradation intermediates of plastics can be directed to form useful by-products. Sufficient education and counseling should be provided to the next generation about the need for removal of plastics from the environments and its proper disposal. Production of polymer blends with biodegradable materials like starch can enhance the degradation process by disrupting the structural integrity and thereby increasing the surface required for the action of enzymes released by the microbes and weakening of mechanical properties of polymers. More interesting and productive research to tap the gut microbial enzymes to degrade plastics and combinatorial use of genomic as well as fermentation techniques could augment the positive results in this direction. The combinatorial use of mechanical methods to plastic remediation to generate microplastics and the microbial methods of bioremediation is yet another promising approach. Moreover, concern should also be raised on pure patenting of plastic degrading enzymes which are of much significance globally in waste remediation.

## Supplementary Material

Supplemental Material
