# Supplementary figures and images for: Bioprospecting of gut microflora for plastic biodegradation
Source: Bioengineered. 2021 Mar 26;12(1):1040–53. doi: 10.1080/21655979.2021.1902173 (PMC8806249; doi:10.1080/21655979.2021.1902173)

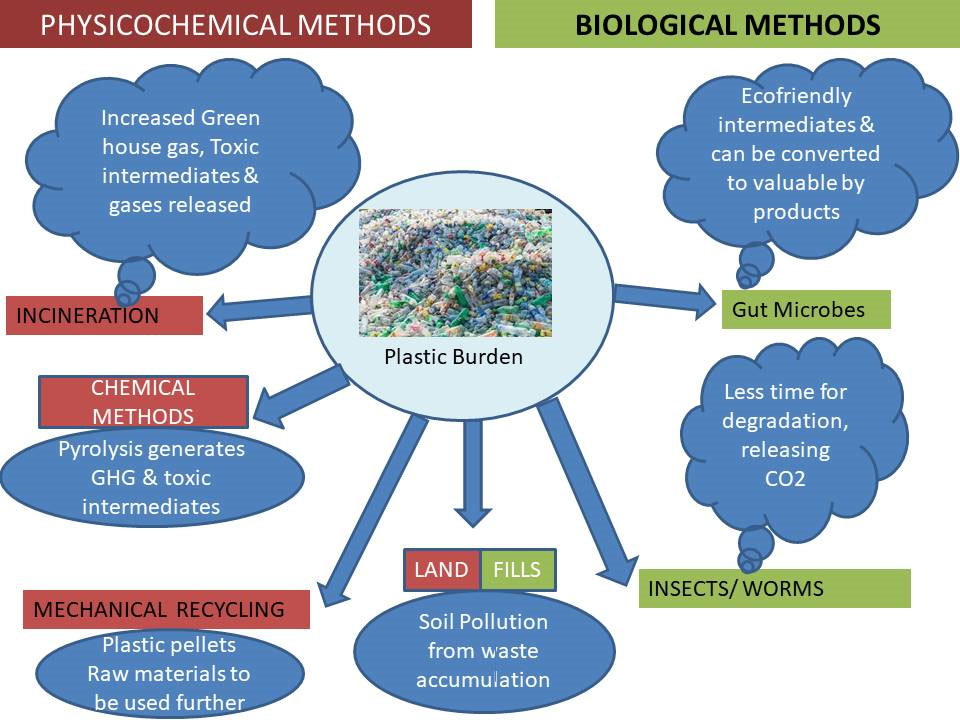

Supplement: Supplemental Material [file KBIE_A_1902173_SM8663.jpg]
